# Leptin-Activity Modulators and Their Potential Pharmaceutical Applications

**DOI:** 10.3390/biom11071045

**Published:** 2021-07-16

**Authors:** Marianna Greco, Marzia De Santo, Alessandra Comandè, Emilia Lucia Belsito, Sebastiano Andò, Angelo Liguori, Antonella Leggio

**Affiliations:** Department of Pharmacy, Health and Nutritional Sciences, University of Calabria, 87036 Arcavacata di Rende, Italy; mariannagreco.89@gmail.com (M.G.); marziadesanto@gmail.com (M.D.S.); alessandracomande@outlook.it (A.C.); emilialucia.belsito@unical.it (E.L.B.); sebastiano.ando@unical.it (S.A.); angelo.liguori@unical.it (A.L.)

**Keywords:** leptin, leptin receptor, leptin-activity modulators, peptide antagonists, leptin mutants, monoclonal antibodies, nanobodies

## Abstract

Leptin, a multifunctional hormone primarily, but not exclusively, secreted in adipose tissue, is implicated in a wide range of biological functions that control different processes, such as the regulation of body weight and energy expenditure, reproductive function, immune response, and bone metabolism. In addition, leptin can exert angiogenic and mitogenic actions in peripheral organs. Leptin biological activities are greatly related to its interaction with the leptin receptor. Both leptin excess and leptin deficiency, as well as leptin resistance, are correlated with different human pathologies, such as autoimmune diseases and cancers, making leptin and leptin receptor important drug targets. The development of leptin signaling modulators represents a promising strategy for the treatment of cancers and other leptin-related diseases. In the present manuscript, we provide an update review about leptin-activity modulators, comprising leptin mutants, peptide-based leptin modulators, as well as leptin and leptin receptor specific monoclonal antibodies and nanobodies.

## 1. Introduction

Leptin is an adipocyte-derived hormone with cytokine-like characteristics that acts both centrally and peripherally. Leptin has a key role in energy metabolism but it is also an important regulator of different physiological and pathological processes, including activation of the immune system and cancer progression [1,2]. It is involved in cancer promoting processes, such as mitogenesis, migration, and invasion. In many human cancer types, it was observed an overexpression of leptin receptor (ObR) highly correlated with leptin presence, suggesting leptin and ObR as important pharmaceutical targets.

Leptin mediates its effects by binding to ObR, therefore, a rational design of leptin modulators requires a deep knowledge of mechanisms driving ObR activation.

Several studies reported the development of compounds that interfere with leptin signaling (such as peptide antagonists, leptin protein mutants, monoclonal antibodies, nanobodies), and, thereby, can be used as potential drugs for the treatment of cancers and other diseases [3,4].

In this review, we present an overview of the different approaches aiming to modulate leptin action including their potential therapeutic use.

## 2. Structure and Binding Sites of Leptin

Leptin is a hormone with a molecular weight of 16 kDa that is encoded by the obesity gene (OB), located on chromosome 7 in humans, that transcribes for a 167 amino acids long peptide with a 21-amino acid signal sequence at the amino-terminus [5,6]. The hormone resulting after the removal of the N-terminal signal peptide is a mature protein of 146 amino acids [7,8]. Leptin is produced primarily, but not exclusively, from adipose tissue, and circulating leptin levels are directly related to percent body fat [9]. Once leptin is secreted in the bloodstream, it crosses the blood–brain barrier and, by binding to its receptor (ObR) in hypothalamic centers [10], controls the energy balance of the body, and keeps energy stores stable [11,12]. Leptin binding to ObR activates several intracellular signaling pathways that are involved in the regulation of different cellular functions. Leptin signaling occurs also in sensory circumventricular organs (CVOs), including the subfornical organ (SFO) and organum vasculosum of the lamina terminalis (OVLT), that lack the normal blood–brain barrier. These organs have fenestrated capillaries that allow circulating leptin the direct access to neurons in this central nervous system region and to act by regulating energy balance and controlling cardiovascular and metabolic function [13,14,15,16,17].

**Figure 1:**
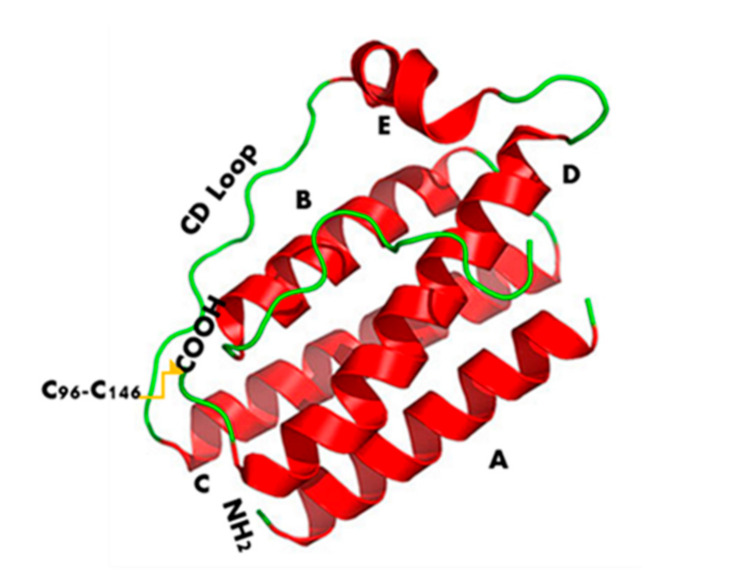
Leptin’s three-dimensional structure. It consists of four antiparallel α-helices (A, B, C, and D), connected by two long crossover links (AB and CD) and one short loop (BC), arranged in up-up-down-down helical bundle. A fifth helix E is contained in the CD loop.

Leptin structure includes four antiparallel α-helices (A, B, C, and D) in an up-up-down-down arrangement, linked by two long crossover links (AB and CD) and one short loop (BC) [18]. A fifth small helix (helix E) is present in the loop linking helices C and D (CD loop) (Figure 1) [19].

**Figure 2:**
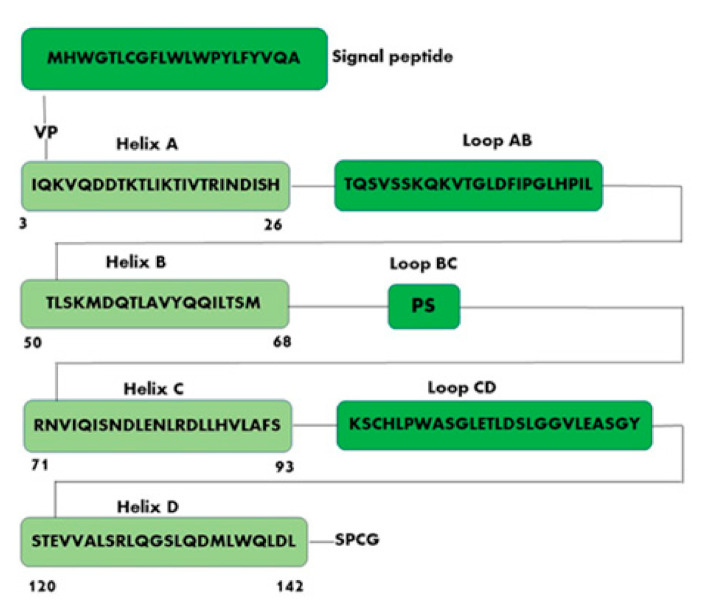
Schematic representation of human leptin sequence. Helix A (amino acid residues 3–26, IQKVQDDTKTLIKTIVTRINDISH); Helix B (amino acid residues 50–68, TLSKMDQTLAVYQQILTSM); Helix C (amino acid residues 71–93, RNVIQISNDLENLRDLLHVLAFS); Helix D (amino acid residues 120–142, STEVVALSRLQGSLQDMLWQLDL).

Leptin sequence (Figure 2) shows numerous hydrophobic residues (Trp100, Phe 92, Leu142, Trp138, and Phe41) responsible for self-association and aggregation of the molecule, some of these are also involved in the receptor binding [20]. Furthermore, leptin has two cysteine residues (C96 in the CD loop and C146 at the C-terminal end) forming a disulfide bridge, crucial for its structural stability and biological activity [20,21]. Amino acid sequence 39–42 in the loop AB (Figure 2) is fundamental for ObR activation and due to this constitutes an important target sequence to be modified for obtaining ObR antagonists [22].

Three different binding sites (I, II and III) are responsible for the interaction of leptin with its receptor. Binding site I plays a key role in the formation of the leptin–leptin receptor complex and in the receptor activation [23], it is formed by the C-terminus of helix D (residues 120–142, STEVVALSRLQGSLQDMLWQLDL) and also contains residues of the connection loop between helix A (residues 3–26, IQKVQDDTKTLIKTIVTRINDISH) and helix B (residues 50–68, TLSKMDQTLAVYQQILTSM). Binding site II consists of residues of helix A and helix C (residues 71–93, RNVIQISNDLENLRDLLHVLAFS); while binding site III includes residues at the N-terminus of helix D (Figure 2).

The importance of binding site I is due to the formation of the hexameric complex composed of two leptin molecules and four leptin receptors [24]. Binding site II is crucial for high affinity binding between leptin and CRH2 domain (cytokine receptor homology domain II) of ObR [25,26], while binding site III interacts with immunoglobulin-like domain (Ig-like domain) of ObR [21] and is involved in the conformational changes for activating ObR [27,28]. However, it remains unclear whether the binding site I is essential for signaling while the location of leptin’s binding site I and binding site III is still controversial [24,25,26,27,28,29].

Leptin exerts its anti-obesity action mainly by targeting ObR-expressing neurons in the hypothalamus in the central nervous system (CNS). Leptin acts on receptors in the lateral hypothalamus to inhibit hunger [30] by counteracting the effects of neuropeptide Y and anandamide, two potent hunger promoters [31]. In the medial hypothalamus, leptin stimulates satiety [30] promoting the synthesis of α-melanocyte-stimulating hormone (α-MSH), a hunger suppressant [32]. Genetic leptin deficiency or lack of functional leptin receptor has been shown to alter brain proteins and neuronal functions of mice and induce morbid obesity and type 2 diabetes which can be reversed by leptin replacement therapy [33]. Instead, high leptin levels, produce atherosclerosis and increase the risk of cardiovascular diseases [34]. However, in many cases, unusually higher levels of circulating leptin are observed in obese patients compared to normal subjects due to the development of leptin resistance that is the reduced leptin ability to suppress hunger and weight gain. Various mechanisms underlying leptin resistance have been proposed, however the key mechanism is the decreased transport of leptin across the blood–brain barrier (BBB) [35].

Leptin can also be produced by tissues other than adipocytes and modulate several physiological processes in the peripheral organs by acting in a paracrine, endocrine as well as autocrine manner [36,37,38,39]. Aside from the adipostatic function, leptin can regulate differentiation and proliferation of hemopoietic cells and macrophage function [40]; produce angiogenesis [41]; increase wound healing [42]; and affect immune and inflammatory responses [43,44]. Leptin and leptin receptor are also involved in the development of various cancers like breast, thyroid, colon, and prostate cancers through pathways that promote proliferation, cell migration and invasion [45,46,47,48,49]. Furthermore, leptin regulates the ovulatory cycle by modulating GnRH release from the hypothalamus [50], it is an important factor in embryo implantation [51], and it is also implicated in the onset of puberty [52].

## 3. Leptin Receptor Structure and Signaling Pathways

Leptin receptor (ObR) is a transmembrane receptor structurally similar to the class I cytokine receptor family [53]. ObR was originally studied via threading analysis, which has revealed that its sequence was compatible with structures from the family of helical cytokines, including the prediction that ObR may activate the JAK–STAT pathway [54]. The forecast was soon demonstrated to be correct when Tartaglia et al. confirmed the identity of the ObR via expression cloning, given its similarity to the interleukin 6 (IL6) signaling receptor chain glycoprotein 130 (gp130), the granulocyte colony-stimulating factor (G-CSF) receptor, and the leukemia inhibitory factor (LIF) receptor [55].

**Figure 3:**
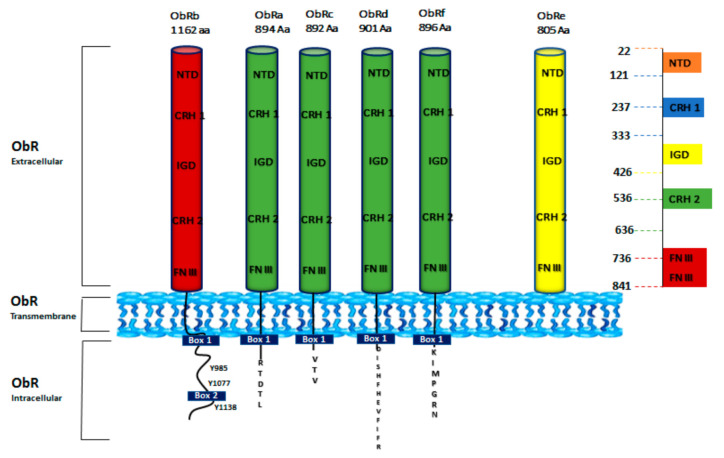
Schematic structure of ObR isoforms. Six isoforms of ObR (ObRa to ObRf) have been identified. ObR is divided into three domains: extracellular (ObR extracellular), transmembrane (ObR Transmembrane), and cytoplasmic tail (ObR Intracellular). All isoforms share the same extracellular domain that consists of an N-terminal domain (NTD), two cytokine receptor homology domains (CRH1 and CRH2), an immunoglobulin-like domain (IGD), and two membrane-proximal fibronectin type III (FN III) domains. The long isoform ObRb has an extended C-terminal domain with three tyrosine residues (Y985, Y1077, and Y1138) [62]. Short forms (ObRa, ObRc, ObRd, and ObRf) have shorter C-terminal regions. ObRe is a soluble variant [63].

ObR is widely expressed in the brain; nevertheless, inferior levels of ObR have been individualized in many peripheral organs [56,57]. ObR presents six isoforms divided into three classes: long (ObRb 1162 Aa), short (ObRa 894 Aa, ObRc 892 Aa, ObRd 901 Aa, and ObRf 896 Aa), and secreted (ObRe 805 Aa) [58]. These isoforms exhibit the same extracellular and transmembrane domain save ObRe, but diverge in the length of their intracellular tail [59]. The extracellular region of all isoforms, composed of 816 amino acids that include four cysteine residues and the WSXWS motif (Trp-Ser-X-Trp-Ser), owns six functional domains: an N-terminal domain (NTD), two CRH domains (CRH1 and CRH2), an immunoglobulin-like domain (Ig-like) and two membrane-proximal fibronectin type III (FN III) domains (Figure 3) [60,61].

CRH1 domain has a regulatory role, while CRH2 is fundamental for leptin binding. The Ig-like domain, indispensable for the receptor conformational change upon ligand binding, and the two FNIII domains are crucial for the receptor activation [64]. The trans-membrane region, consisting of 34 amino acids, includes the long isoform ObRb and the short ones-ObRa, ObRc, ObRd, and ObRf, while ObRe is not membrane-connected [65]. Leptin receptor isoforms have different lengths of their intracellular C-terminal domain: the intracellular domain of ObRb is the longest and contain 303 amino acids, while those of the short isoforms include 32–40 amino acids. The first intracellular 29 amino acids proximal to the transmembrane region show the same sequence for the short and long isoforms. ObRe isoform is secreted to the bloodstream as a soluble receptor [66,67] and lacks both transmembrane and cytoplasmic domains. The intracellular sequence is made up of a constant Box1 motif that is required for Janus kinase (JAK) interaction and activation. Box1 and Box2 (contained only in ObRb isoform) motifs are known to recruit and bind JAKs. It was reported that JAK2 is the only JAK kinase associated with ObRb intracellular domain to mediate intracellular signaling. ObRb presents three highly conserved tyrosine residues (Y985, Y1077, Y1138) essential for efficient leptin signaling [67,68]. The other forms lack some or all of these motifs (Figure 3).

**Figure 4:**
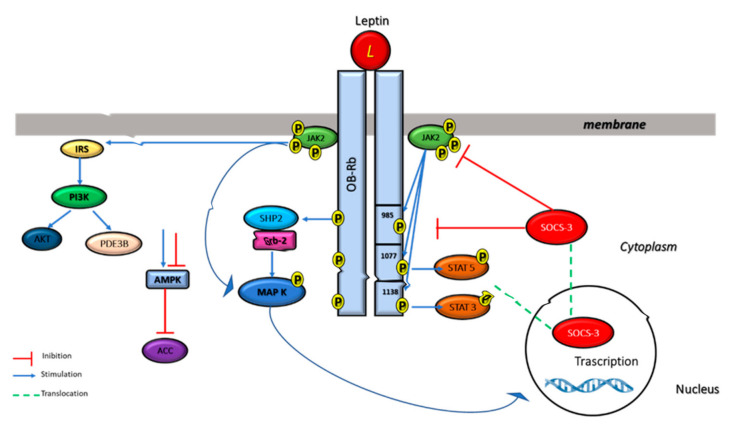
Multiple leptin signaling pathways. Leptin binding to its receptor activates the JAK–STAT3, PI3K, and ERK pathways. For JAK–STAT activation, activated JAK2 tyrosine kinase induces the phosphorylation of Tyr985 and Tyr1138 of ObRb, which results in the activation of STAT3/STAT5. Phospho-Tyr985 recruits SHP-2 and GRB2 resulting in the activation of ERK signaling. Furthermore, leptin activates PI3K by recruiting IRS proteins and it is also able to inhibit AMPK activity.

High levels of ObRb are expressed in ventromedial and lateral hypothalamus, regions of the brain involved in the regulation of body weight [69]. The short isoforms are expressed in the choroid plexus and brain microvessels, structures that constitute the blood-cerebrospinal fluid and blood–brain barriers, respectively, suggesting a role in mediating the brain uptake of leptin [70]. The ObRe isoform in humans is generated by proteolytic ectodomain shedding [71], and is involved in modulating leptin’s biological activity [72]. Through its receptors, leptin plays important roles in several cellular functions throughout the body. Leptin-receptor binding induces the activation of a number of intracellular signaling pathways, such as Janus-activated kinase 2 (JAK2)/signal transducer and activator of transcription 3 (STAT3), insulin receptor substrate (IRS)/phosphatidylinositol 3 kinase (PI3K), SH2-containing protein tyrosine phosphatase 2 (SHP2)/mitogen-activated protein kinase (MAPK), and 5′ adenosine monophosphate-activated protein kinase (AMPK)/acetyl-CoA carboxylase (ACC) [73] (Figure 4).

ObRb is the isoform of the leptin receptor that is primarily responsible for leptin signaling [74]. Leptin induces JAK2 activation which leads to the phosphorylation of different tyrosine residues of the ObR, including Tyr985, Tyr1077, and Tyr1138. The phosphorylated tyrosine residues bind to downstream molecules and activate the JAK2/STAT3, JAK2/STAT5, PI3K/IRS/AKT, and SHP2/ERK pathways [75,76].

Phosphorylated Tyr 1138 binds to the Src homology 2 (SH2) domain of STAT3 proteins allowing phosphorylated STAT3 to enter the nucleus and to regulate the transcription of their target genes, including suppressor of cytokine signaling 3 (SOCS3) (Figure 4), while the phosphorylation of Tyr 1077 on the docking site for the Src homology 2 (SH2) activates STAT5. JAK/STAT3/5 pathways seems to have a key role in energy homeostasis and neuroendocrine function [76,77,78,79,80]. Leptin also regulates MAPK signaling pathway via tyrosine phosphorylation of JAK2 receptor-associated activation, or regardless of receptor phosphorylation.

The phosphorylation of Tyr985 activates signaling related with SH2-containing protein tyrosine phosphatase 2 (SHP2), leading to the activation of the extracellular signal-related kinase (ERK) signaling pathway via growth factor receptor binding-2 (GRB-2), which, in turn, controls the expression of specific target genes, such as c-fos, jun, and egr-1, fundamental in cell proliferation and differentiation [77,81,82,83]. SHP2/ERK pathway, activated by phospho-Tyr985, controls also leptin’s anti-obesity function. SOCS3 competes with SHP2 for the phospho-Tyr985 and progressively reduces the SHP2-ERK pathway [84] (Figure 4). Consistent with this hypothesis, it was reported that the replacement of Tyr985 with Phe promotes diet-induced leptin resistance and obesity [85].

Leptin also promotes the activation of phosphatidylinositol 3 kinase (PI3K) pathways via insulin receptor substrate (IRS) phosphorylation. Leptin acts through some insulin receptor substrates (IRSs) that, after phosphorylation, induce PI3K activation, indicating a direct and important cross-talk between the insulin and leptin signaling pathways [66]. PI3-kinase products activate Akt, also called protein kinase B (PKB), a serine/threonine kinase that has a crucial role in many cellular processes, such as carbohydrate metabolism [86,87]. Leptin enhances glucose uptake and metabolism also by activating of 5′-AMP-activated protein kinase (AMPK), an enzyme that controls cellular energy status.

Leptin, by activating AMPK and blocking ACC (acetyl-CoA carboxylase) activity, stimulates fatty acid oxidation, thereby protecting non-adipose tissue against lipotoxicity [88,89].

## 4. Leptin-Activity Modulators

In the area of medicinal chemistry, a wide repertoire of leptin-related antagonists and agonists have been developed [90,91].

The leptin involvement in body weight regulation, immunity, angiogenesis, and cancers [38,72,92] has generated a great interest in the design and development of different leptin and ObR-based therapeutic approaches. Leptin mutants, leptin peptide antagonists, and neutralizing antibodies, have been shown to possess a promising therapeutic potential for the treatment of various diseases, including cancers [90,93,94].

Elevated levels of serum leptin have been unequivocally correlated to an increased risk of developing various tumor forms: testicular, breast, prostate, colon, and pancreatic cancers [46,95,96,97]. Several studies have reported that leptin and leptin receptor are implicated in cancer, mainly via the JAK/STAT pathway, which regulates PI3K/AKT3 and ERK1/2 signaling, angiogenic factors (VEGF), systemic inflammation (TNF-α, IL6), and anti-apoptotic proteins (XIAP) expression [98]. Leptin alters the tumor microenvironment and favors tumor growth and progression by stimulating migrations of endothelial cells and angiogenesis [99,100], suppressing apoptosis and assisting the recruitment of macrophages and monocytes, which secrete vascular endothelial growth factor [101] and proinflammatory cytokines.

Blocking of leptin activity is, therefore, particularly appropriate in these pathological situations. Antagonist peptides block leptin signaling by preventing the association of the leptin receptor with the endogenous ligand. Among them, there are antagonistic mutants and leptin fragments, able to bind the receptor without activating it, as well as leptin receptor-specific (monoclonal) antibodies or nanobodies that prevent leptin from binding.

### 4.1. Leptin Mutants

The first developed leptin antagonist was a modified form of human leptin containing the mutation Arg128Gln [102]. The replacement of glutamine at position 128 of human leptin with an arginine residue abolished the biological activity on ObR expressing Ba/F3 cells without affecting the binding to the ObR.

It has been reported that the residues D9, T12, K15, T16, and R20 located on helix A and Q75, N82, D85 and L86 located on helix C are structurally close and, most likely, are involved in the interaction with CRH2 domain of ObR [28]. Mutations in these sites, such as D9S, T12Q, K15S, T16N, R20N, Q75S, N82S, D85S and L86A, L86S, L86N, and L86Q, significantly affected binding to CRH2 but had a limited effect on ObR signaling [23,26].

Mutations on the binding site III of leptin at the N-terminus of helix D significantly inhibit receptor activation without altering binding to CRH2. These muteins behave as typical leptin antagonists. They bind to ObR with an affinity similar to that of native leptin, and have no agonistic activity.

**Figure 5:**
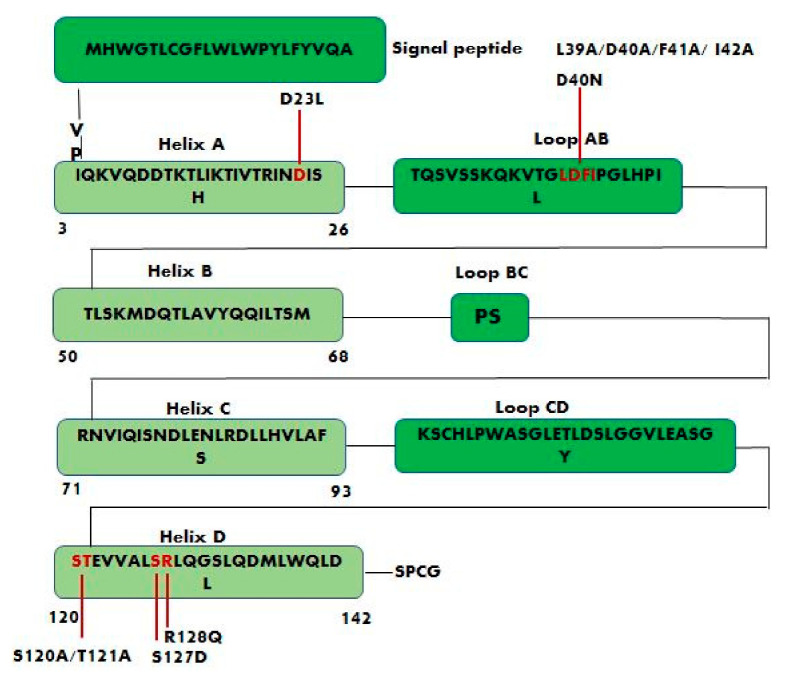
Schematic representation of leptin sequence showing the positions of the main studied mutations. Leptin antagonists L39A/D40A/F41A, L39A/D40A/F41A/I42A and S120A/T121A are the result of alanine mutagenesis of residues LDF (amino acids 39–41), LDFI (amino acids 39–42), and ST (amino acids 120–121) respectively. D23L, D40N, S127D, and R128Q are other important mutations that affect leptin biological activity.

The LDFI sequence (amino acids 39–42) located in the loop connecting helices A and B of leptin contributes to activate leptin receptor. The sequence 39–42 interacts with the amino acids 325–328 (^325^VFTT^328^) of the receptor IgD domain. The mutations in the sequence 39–42 of some or all amino acids produced leptin muteins able of binding to the receptor, but unable to bring about ObR activation [29]. The replacement of the amino acids of the sequence 39–42 with alanine residues resulted in the creation of leptin antagonists: the triple mutein L39A/D40A/F41A (LDF or Lan-1) and the quadruple mutein L39A/D40A/F41A/I42A (LDFI or Lan-2) [103] (Figure 5). The bioavailability of these antagonists was enhanced by pegylation and resulting in potent antagonists in vivo.

Salomon G. et al. developed leptin antagonists in their laboratory and found that the mutation D23L greatly enhanced the affinity of human leptin toward the leptin receptor [104]. The S120A/T121A leptin mutant in which Ser120 and Thr121, located on the N-terminus of helix D (binding site III), are replaced by the amino acid alanine (Figure 5), behaves as an antagonist and, in fact, is able to bind selectively to the CRH2 domain of ObR without causing its activation. The treatment of mice with S120A/T121A leptin mutant caused an increase in body weight indicating that the leptin antagonist stimulates food intake [23]. Similarly, the leptin antagonist R128Q, that is not part of any of the three predicted binding sites, increases the body weight in mice and probably affects binding site III indirectly by modifying the proper orientation of the AB and CD loops [102]. Additionally, the mutations D40N and S127D (Figure 5) did not modify receptor binding, but they affected leptin biological activity [105]. The binding to the receptor is dominated by the interaction of leptin residues L86 and L13 (binding site II) with L504 of the CRH2 domain [106].

The combination of the D23L mutation with alanine mutagenesis of residues LDF (L39A/D40A/F41A) produced a D23L/L39A/D40A/F41A mutant, a potent leptin antagonist called super-active human leptin antagonist (SHLA). SHLA has 14-fold higher antagonistic activity as compared with the leptin antagonist L39A/D40A/F41A showing 3 mutations [107]. SHLA, blocking the leptin receptor, can reverse leptin action in ovarian follicles, and, consequently, and can also be useful for controlling fertility [108]. The effects of the two leptin receptor antagonists, super-active human leptin antagonist (SHLA) and quadruple leptin mutein Lan-2 (L39A/D40A/F41A/I42A), on cell proliferation and progression of epithelial ovarian tumors were also evaluated [109]. The study was conducted on three different cell lines: serous carcinoma (OVCAR-3), metastatic carcinoma (CaOV-3) and non-cancerous human ovarian surface epithelial cells (HOSEpiC), showed that SHLA and Lan-2 are promising leptin receptor inhibitors that can be used to block the progression of epithelial ovarian cancers.

Fiedor E. et al. also studied the effects of the leptin receptor antagonists, SHLA, Lan-2, and Lan-1 (L39A/D40A/F41A) on the progression of human folliculoma and, in particular, on two cell lines: COV434 (the juvenile form of granulosa tumor), and KGN (the adult type corresponding to peri- to postmenopausal age). In KGN cells, all three ObR antagonists reversed leptin-stimulated proliferation at all concentrations via interactions with cell cycle protein expression. These findings suggested that these muteins could be considered promising candidates to abolish the negative effects of leptin. On the contrary, the effects of these antagonists were negligible in the COV434 cell line [110].

The overexpression of histone deacetylases (HDACs), critical regulators of gene expression, was found in many types of cancers and is associated with accelerated cell proliferation. HDAC inhibitors are used as antineoplastic drugs and showed encouraging efficacy in cancer patients. Considering that leptin affects HDACs levels, the effect of leptin receptor antagonists on HDAC expression was evaluated [111]. This study, conducted in ovarian epithelial (OVCAR-3, CaOV3) and folliculoma (COV434, KGN) cells, has demonstrated that SHLA and Lan-2 reversed the stimulatory effect of leptin on the HDACs in a cell-type-dependent manner and, therefore, they could show beneficial effects on the treatment of ovarian cancer.

### 4.2. Peptide-Based Leptin Receptor Antagonists

Peptide-based leptin modulators are short chain peptides consisting of parts of the original leptin sequence, they can act as antagonists or agonists [90,112]. Peptide antagonists of the leptin receptor (ObR) have been developed to block the pro-neoplastic leptin action, both in vitro and in vivo. The use of leptin receptor antagonists has been extensively studied for the treatment of breast cancer. In breast cancer cells, short and long ObR isoforms are overexpressed [113] and leptin has been shown to induce several signaling pathways and factors linked to the proliferation, migration, and invasion of cancer cells.

**Figure 6:**
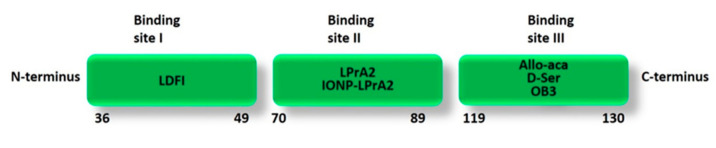
Schematic representation of the main leptin peptide analogues with anti-tumor activity based on human leptin binding sites. LDFI is based on the sequence 39–42 (LDFI) of leptin binding site I; LPrA2 and IONP-LPrA2 are based on the amino acid sequence 70–95 of leptin binding site II; Allo-aca (H-alloThr-Glu-Nva-Val-Ala-Leu-Ser-ArgAca-NH_2_) and D-Ser (H-(D)Ser-Glu-Nva-Val-Ala-Leu-Ser-(N-Me)Arg-βAla-NH_2_) are based on the modified amino acid sequence 121–129 of leptin binding site III; OB3 is based on the amino acid sequence 116–122 of leptin binding site III.

The first leptin peptide analogues with antagonistic activity towards leptin receptor and efficient anti-tumor activity in breast cancer were designed based on leptin binding sites II and I [114,115] (Figure 6). LPrA-2, a fragment containing leptin site II and corresponding to amino acids 70–95 of human leptin, binds specifically and with high affinity to ObR and, acting as a leptin antagonist, inhibits leptin-related effects in breast cancer and other cancer types [115].

The pegylated form of this antagonist, PEG-LPrA2, attenuated the expression of vascular endothelial growth factor (VEGF) and vascular endothelial growth factor receptor type 2 (VEGFR2) in MCF-7 (ER + human breast cancer cells) and MDA-MB-231 (ER-human breast cancer cells) breast cancer in mice [114]. LPrA2 showed a higher efficacy in reducing tumor growth in ER + MCF-7 than in ER- MDA-MB-231 breast cancer xenografts, this probably depends on the greater ObR expression in the MCF-7 model.

Recently, Gonzalez-Perez and colleagues determined the efficacy of leptin receptor antagonists associated with nanoparticles. They developed a leptin antagonist nanosystem (IONP-LPrA2) based on iron oxide nanoparticles (IONP) linked to the antagonist LPrA2. IONP-LPrA2 markedly decreased leptin-induced proliferation of breast cancer cells treated with chemotherapeutics [116]. The results of this study indicated that IONP-LPrA2 represents an efficient delivery system for the leptin antagonist LPrA2 to target and treat breast cancer; furthermore, it acts as an adjuvant when administered with chemotherapeutics. The leptin antagonist, IONP-LPrA2, was also used to reduce leptin-induced effects in pancreatic cancer (PC) cells [117].

Otvos et al. developed leptin peptide analogues based on the binding sites I (peptide 36–49), II (combined construct 3-18-GGG-70-89), and III (peptide 117–132) and evaluated their effect on the proliferation of ObR-positive and ObR-negative cells lines [118]. These leptin peptides showed antagonist and agonist properties depending on the presence of native leptin. The site III derivative 117–132 mutated with two alanine at positions 120 and 121 proved to be a strong leptin antagonist; however, in absence of leptin, it showed agonistic activity since it activated cell growth. Similarly, the combined site II construct blocked leptin-induced growth, but when it was tested in the absence of the hormone, it proved to be a full agonist. Instead, the site I-based peptide, both with and without native leptin addition, did not significantly affect the cell growth in MCF-7 cells (ObR-positive).

Allo-aca, a 9-amino acid-long peptide (H-alloThr-Glu-Nva-Val-Ala-Leu-Ser-ArgAca-NH_2_, Aca: 6-amino-caproic acid), is another peptide analogue of leptin binding site III (Figure 6) that acts as a potent antagonist without showing any partial agonist activity. It was able to inhibit leptin-dependent growth of human breast cancer xenografts by approximately 50% when administered intraperitoneally or subcutaneously at a 0.1 mg/kg/day dose. In a model triple-negative breast cancer cell line MDA-MB-231, the peptide antagonist Allo-aca at 1 pM–100 nM concentrations proved to antagonize the mitogenic effects of leptin in a concentration-dependent manner without interfering with MDA-MB-231 proliferation in the absence of native leptin [119,120]. The considerable in vivo efficacy data are related to the extreme stability of the complex formed by the antagonist peptide with the receptor demonstrating the high binding affinity between Allo-aca and ObR. Allo-aca dissociates from the complex slower than the full-length leptin, although Allo-aca uses only one of the three ObR-binding sites [121].

On the base of Allo-aca structure, other ObR peptide antagonists were designed and developed featuring exclusively peripheral activity, in order to avoid the undesirable CNS effects of Allo-aca, such as moderate weight gain in some in vivo models. The anti-neoplastic activity and biodistribution of the Allo-aca analogues were assessed in breast and colon cancer cells in vitro. The analogue D-Ser (H-(D)Ser-Glu-Nva-Val-Ala-Leu-Ser-(N-Me)Arg-βAla-NH_2_), incorporating in the peptide chain a D-amino acid to prevent BBB penetration, efficiently inhibited leptin-induced growth in colon adenocarcinoma HT29 and breast cancer MCF7 cells at 1 nM concentration without any partial agonistic activity in the absence of the exogenous leptin [122].

The amino acid sequence 39–42 (LDFI), crucial for activating leptin receptor, represents a key region for designing potential ObR antagonists [23]. Based on the wild-type sequence of leptin binding site I, a leptin receptor peptide antagonist consisting of the short chain peptide LDFI has been synthesized by solid phase peptide synthesis [123].

The LDFI peptide acted as a full leptin antagonist by inhibiting the leptin-induced growth and migration in both ERα-positive (MCF-7) and ERα-negative (SKBR3) breast cancer cells without interfering with cell proliferation in the absence of leptin. LDFI anticancer activity was correlated to the inhibition of leptin-induced JAK2, STAT3, AKT, and MAPK pathways, and the reduction in Cyclin D1 expression [123]. The more stable and bioavailable pegylated form of the LDFI peptide (LDFI-PEG) was also synthesized to assess the efficacy of the peptide in mouse xenograft models. LDFI-PEG significantly reduced breast tumor growth in SKBR3 xenografts.

Leptin, as a mediator of tumor-stromal interactions, can affect the activity of breast cancer stem cells (BCSC) that play a key role in tumor progression. The inhibition of leptin signaling to reduce BCSC activity was studied in vitro and ex vivo models by using the leptin receptor antagonist peptide LDFI. This study revealed that LDFI markedly inhibited mammosphere formation in metastatic breast cancer patient-derived cells, demonstrating how the inhibition of leptin signaling might represent an efficient approach to block cancer progression mediated by BSCM [124].

In a recent study, it was observed that the expression of leptin and its receptor in human testicular seminoma is markedly higher than in normal adult testis. Furthermore, an enhanced proliferation and migration of human seminoma cells, TCam-2, following leptin treatment was reported. In the light of these data, the leptin-receptor antagonist LDFI, was tested on TCam-2 cells in order to evaluate the effects of inhibiting leptin signaling on testicular cancer growth and progression. The peptide Leu-Asp-Phe-Ile (LDFI), totally reversed the leptin-induced effects on cell growth and motility, also the in vivo experiments proved that the pegylated LDFI (LDFI-PEG) dramatically decreased the growth of TCam-2 xenografts [95].

LDFI, as inhibitor of leptin activity, has been also used for the identification of the signals involved in exosome biogenesis in breast cancer cells and to elucidate the mechanisms driving cancer exosome production. LDFI peptide significantly reduced exosome secretion in both MCF-7 and MDA-MB-231 breast cancer cells, demonstrating the leptin/leptin receptor/Hsp90 axis as an important regulator of exosome generation in mammary carcinoma cells [125].

More recently, the inhibition of leptin signaling by LDFI confirmed the involvement of the leptin-mediated signaling pathway in the development of aromatase inhibitor (AI) therapy resistance in breast cancer [126]. In addition, LDFI showed to be very effective in abrogating the leptin-promoted proliferation and migration of human glioblastoma cells, U-87 MG and T98G, and in reducing the expression of many leptin-induced target genes [127].

A new class of functional leptin peptides (OB3) corresponding to amino acid residues 116–130 of mouse leptin has been proposed by Grasso and co-workers. The activity of OB3-leptin peptide is correlated to the N-terminal residues 116–122 (Ser-Cys-Ser-Leu-Pro-Gln-Thr, OB3). Furthermore, the replacement of Leu residue with the corresponding d-isomer ([d-Leu-4]-OB3) improved the OB3 antihyperglycemic action [128]. The signaling pathways and gene expressions modulated by leptin and OB3 were investigated in different cancer cell lines. OB3 peptide did not promote cell growth in ovarian cancer cells, furthermore, it inhibited leptin-induced proliferative signals when it was used in combination with leptin [129]. Similarly, OB3 peptide did not show the leptin mitogenic effects on thyroid cancer cells. The in vivo studies also proved that leptin, but not OB3, remarkably enhanced the levels of thyrotropin (TSH), a growth factor for thyroid cancer [130].

In a recent study Lin et al. investigated the anti-inflammation and anti-proliferation activity of the leptin-peptides OB3 and [D-Leu-4]-OB3 in two hepatocellular cancer (HCC) cell lines, HepG2 and Hep3B. Results showed that both OB3 and [D-Leu-4]-OB3 suppressed the expression of pro-inflammatory, proliferative and metastatic genes in HCC cell lines and activated the expression of pro-apoptotic genes. OB3, in the presence of leptin, blocked leptin-promoted proliferation in HCC cells by suppression of phosphoinositide 3-kinase (PI3K) activation [131].

In another study, Grasso and co-workers reported that the peptide [D-Leu-4]-OB3 and its myristic acid conjugate MA-[D-Leu-4]-OB3, cross the blood–brain barrier and accumulate in a region of the hypothalamus that regulates energy balance and glucose homeostasis, indicating that these peptides act to stimulate leptin receptors in this area [132]. The same research group in a later work observed that MA-[D-Leu-4]-OB3 improves episodic memory in mouse models. This ability is due to the glycemic control as well as to the reduction of pro-inflammatory cytokines involved in neuronal degeneration [133]. Therefore, MA-[D-Leu-4]-OB3 could represent a suitable therapeutic agent for the treatment of Alzheimer’s disease.

### 4.3. Antibodies and Nanobodies

Leptin activity can also be inhibited by specific anti-leptin-receptor monoclonal antibodies (anti-LR mAbs) that, in binding to the leptin receptor, suppress leptin signaling. Due to their high molecular mass, they present the advantage of a long circulating half-life, furthermore, they have a good affinity for the receptor.

In 2006, Fazeli et al. identified a leptin-blocking monoclonal antibody (mAb), 9F8, which prevents the binding of leptin to ObR, thereby acting as a leptin antagonist in vitro [134]. The specific mAb, 9F8, reduced leptin-induced tumor necrosis factor α (TNF-α) expression by human monocytes, and the proliferation of peripheral blood mononuclear cells (PBMC). In a later study, the effect of mAb, 9F8, on leptin-induced cell viability and proliferation of ERα-positive (MCF7) and ERα-negative (MDA-MB 231) human breast cancer cell lines was analysed. The leptin-mediated strong phosphorylation of STAT3 and ERK1/2 was blocked in the presence of mAb 9F8 in MCF 7 cells only, thus confirming the presence of leptin receptor in the ERα-positive cell lines [135]. This antibody antagonizes leptin binding through a partial overlap in their binding sites (10%) [136].

Another group used the monoclonal antibody 9F8 Fab to design peptidomimetics with anti-leptin activity by computational approaches. They studied a model of the leptin receptor bound to leptin and 9F8 Fab and performed protein–protein docking to investigate the interface interactions and the involved amino acids. Six small peptide antagonists for ObR were proposed that showed better binding affinity in docking and molecular dynamics simulations compared to native 9F8 Fab peptides [137].

A recent study demonstrated that the administration of both polyclonal and monoclonal anti-leptin receptor antibodies in growing chickens promoted leptin-like effects such as the upregulation of phosphorylated STAT3 protein in the liver and the reduction in plasma glucose levels increasing feed intake [138]. However, these antibodies proved to increase and not mimic the ability of leptin to activate the receptor, since they did not promote leptin receptor signaling when administered in absence of leptin.

Recently, Tavernier et al. reported a camelid single-domain antibody that acts as a leptin receptor antagonist by selectively inhibiting the cross-talk between leptin receptor (LR) and the epidermal growth factor receptor (EGFR). This antibody interferes with the leptin immunomodulatory effects but not with its metabolic functions. This study showed that the structural requirements for LR signaling and LR-EGFR cross-talk are different. On this base, a VHH (variable domain of heavy chain antibody) able to block the cross-talk but not the LR signaling (through leptin site III–IGD interaction) that controls metabolism and body weight has been proposed [139].

The disadvantages of full-length mAbs, including large size, poor tissue penetration, and possible immunogenicity, led to the development of monomeric, small neutralising nanobodies targeting the leptin receptor and blocking leptin signaling. Nanobodies (Nb) are small single domain antigen-binding fragments (~15 kDa), derived from heavy-chain-only antibodies present in camelids. They have high stability, strong antigen-binding affinity and can selectively block the leptin peripheral activity since they do not generally cross the blood–brain barrier [140].

Zabeau et al. developed a series of neutralizing nanobodies targeting the leptin receptor and selected them on the basis of their interaction with different leptin receptor subdomains: CRH2, IGD, and FNIII domains. They showed that only the nanobodies that interacted with the CRH2 domain were able to inhibit leptin binding to its receptor, being CHR2 the main leptin-binding determinant, while the nanobodies that targeted the IGD and FNIII domains blocked the signaling without affecting leptin binding. All the three selected nanobodies, 2.17-mAlb (against CHR 2, interfering with leptin binding), 4.10-mAlb (against IGD), and 4.11-mAlb (against FNIII), interfered with leptin-induced JAK2 phosphorylation and STAT3 activation. These results proved, also, that it is possible to inhibit leptin receptor signaling without affecting leptin binding [141].

The anticancer activity of 2.17-mAlb was assessed in a mouse model of melanoma. The local administration adjacent to tumor of low-dose 2.17-mAlb remarkably slowed the melanoma growth and reduced melanoma mass by 33% without affecting weight and food intake showing that the central action of leptin was not blocked [142]. On the contrary, the systemic administration of high dose 2.17-mAlb produced the suppression of the central action of leptin resulting in weight gain and reduced the anticancer effect of the nanobody. These results demonstrated that peripherally acting peptides are more efficient leptin antagonists.

The unique structural and functional properties of nanobodies make them highly attractive therapeutic agents for targeting important angiogenic factors involved in tumorigenesis as leptin [143].

The leptin modulators and their anticancer activity are schematically reported in Table 1.

## 5. Conclusions

Leptin and leptin receptor play an important role in body weight maintenance and energy homeostasis. Leptin’s involvement in many peripheral biological functions, as well as in autoimmune diseases and cancer increased the interest for the design and development of leptin-based drugs acting as leptin-activity modulators.

The studies reported in this review cover the main developed strategies to date to modulate leptin signaling including leptin muteins, peptide-based leptin receptor antagonists, neutralizing antibodies and nanobodies.

The majority of the reported molecules act as ObR antagonists and are mutants of the full native protein (SHLA, Lan-2, and Lan-1) and peptide fragments (LPrA2, Allo-aca, and D-ser, LDFI) relating to single receptor-binding site. Although these strategies were demonstrated as quite efficient in various cancer models, in some cases they resulted in a significant unwanted weight gain in mice upon treatment.

The ability of the leptin receptor to interact with other receptor systems (epidermal growth factor receptor (EGFR), estrogen receptor alpha (ERα), insulin-like growth factor I receptor (IGF-IR)) may offer the opportunity to overcome this limit by uncoupling leptin’s central and peripheral functions and to design more selective leptin receptor antagonists, with an improved therapeutic action.

All these findings might contribute to a better knowledge of the complex biological mechanisms responsible for leptin activities and pave the way for the future development and clinical application of novel and more effective leptin receptor modulators able to prevent the undesired metabolic side effects.

## Figures and Tables

**Table 1:** The main leptin modulators with anticancer activity.

KERRYPNX	Designation	Cancer Type	In Vitro Studies	In Vivo Studies
Muteins	L39A/D40A/F41A (Lan-1)	Human Folliculoma	[110]	
	L39A/D40A/F41A/I42A (Lan-2)	Ovarian Tumor	[109,111]	
		Human Folliculoma	[110,111]	
	D23L/L39A/D40A/F41A (SHLA)	Ovarian Tumor	[109,111]	
		Human Folliculoma	[110,111]	
Peptides	LPrA-2	Breast Cancer	[114]	[114]
		Endometrial Cancer	[115]	
	IONP-LPAr2	Breast Cancer	[114]	
		Pancreatic cancer	[117]	[117]
	Allo-aca	Breast Cancer	[119,120,121]	[119,120,121]
	D-Ser	Breast Cancer	[122]	
		Colorectal Cancer	[122]	
	LDFI	Breast Cancer	[123,124]	[123]
		Seminoma	[95]	[95]
		Glioblastoma	[127]	
	OB3	Ovarian Cancer	[129]	[129]
		Hepatocellular Cancer	[131]	
Antibodies	9F8	Breast Cancer	[135]	
Nanobodies	2.17-mAlb	Melanoma	[142]	[142]

## Data Availability

Not applicable.

## References

[1] Samad N., Rao T. (2019). Role of leptin in cancer: A systematic review. Biomed. J. Sci. Tech. Res..

[2] Ramos-Lobo A.M., Donato J. (2017). The role of leptin in health and disease. Temperature.

[3] Surmacz E. (2013). Leptin and adiponectin: Emerging therapeutic targets in breast cancer. J. Mammary Gland Biol. Neoplasia.

[4] Scolaro L., Cassone M., Kolaczynski J.W., Otvos L., Surmacz E. (2010). Leptin-based therapeutics. Expert Rev. Endocrinol. Metab..

[5] Zhang F., Basinski M.B., Beals J.M., Briggs S.L., Churgay L.M., Clawson D.K., DiMarchi R.D., Furman T.C., Hale J.E., Hsiung H.M. (1997). Crystal structure of the obese protein leptin-E. Nature.

[6] Wallace A.M. (2000). Measurement of leptin and leptin binding in the human circulation. Ann. Clin. Biochem..

[7] Friedman J.M. (2010). A tale of two hormones. Nat. Med..

[8] Zhang Y., Proenca R., Maffei M., Barone M., Leopold L., Friedman J.M. (1994). Positional cloning of the mouse obese gene and its human homologue. Nature.

[9] Frederich R.C., Hamann A., Andersn S., Löllman B., Lowell B.B., Flier J.S. (1995). Leptin levels reflect body lipid content in mice: Evidence for diet-induced resistance to leptin action. Nat. Med..

[10] Schwartz M.W., Wood S.C., Porte D., Seeley R.J., Baskin D.G. (2000). Central nervous system control of food intake. Nature.

[11] Friedman J.M., Halaas J.L. (1998). Leptin and the regulation of body weight in mammals. Nature.

[12] Banks W.A. (2001). Leptin transport across the blood-brain barrier: Implications for the cause and treatment of obesity. Curr. Pharm. Des..

[13] Young C.N., Morgan D.A., Butler S.D., Mark A.L., Davisson R. (2013). The brain subfornical organ mediates leptin-induced increases in renal sympathetic activity but not its metabolic effects. Hypertension.

[14] Smith P.M., Chambers A.P., Price C.J., Ho W., Hopf C., Shankey K.A., Ferguson A.V. (2008). The subfornical organ: A central nervous system site for actions of circulating leptin. Am. J. Regul. Integr. Comp. Physiol..

[15] Hindmarch C.C.T., Ferguson A.V. (2016). Physiological roles for the subfornical organ: A dynamic transcriptome shaped by autonomic state. J. Physiol..

[16] Smith P.M., Ferguson A.V. (2011). Cardiovascular actions of leptin in the subfornical organ are abolished by diet-induced obesity. J. Neuroendocrinol..

[17] Reddy V.D.K., Jagota A. (2014). Effect of restricted feeding on nocturnality and daily leptin rhythms in OVLT in aged male Wistar rats. Biogerontology.

[18] Zabeau L., Peelman F., Tavernier J. (2015). Leptin: From structural insights to the design of antagonists. Life Sci..

[19] Denver R.J., Bonett R.M., Boorse G.C. (2011). Evaluation of leptin structure and function. Neuroendocrinology.

[20] Zhang F., Chen Y., Heiman M., Dimarchi R. (2005). Leptin: Structure, function and biology. Vitam. Horm..

[21] Rock F., Altmann S.W., van Heek M., Kastelein R.A., Bazan J.F. (1996). The liptin haemopoietic cytokine fold is stabilized by an intrachain disulfide bond. Horm. Metab. Res..

[22] Gertler A. (2006). Development of leptin antagonists and their potential use in experimental biology and medicine. Trends Endocrinol. Metab..

[23] Peelman F., van Beneden K., Zabeau L., Iserentant H., Ulrichts P., Defeau D., Verhee A., Catteeuw D., Elewaut D., Tavernier J. (2004). Mapping of the leptin binding sites and design of a leptin antagonist. J. Biol. Chem..

[24] Peelman F., Iserentant H., De Smet A.-S., Vandekerckhove J., Zabeau L., Tavernier J. (2006). Mapping of binding site III in the leptin receptor and modeling of a hexameric leptin· leptin receptor complex. J. Biol. Chem..

[25] Fong T.M., Huang R.R., Tota M.R., Mao C., Smith T., Vernerin J., Karpitskiy V.V., Krause J.E., Var der Ploeg L.H. (1998). Localization of leptin binding domain in the leptin receptor. Mol. Pharm..

[26] Iserentant H., Peelman F., Defeau D., Vandekerckhove J., Zabeau L., Tavernier J. (2005). Mapping of the interface between leptin and the leptin receptor CRH2 domain. J. Cell. Sci..

[27] Zabeau L., Lavens D., Peelman F., Eyckerman S., Vandekerckhove J., Tavernier J. (2003). The ins and outs of leptin receptor activation. FEBS Lett..

[28] Peelman F., Couturier C., Dam J., Zabeau L., Tavernier J., Jockers R. (2006). Techniques: New pharmacological perspectives for the leptin receptor. Trends Pharm. Sci..

[29] Niv-Spector L., Gonen-Berger D., Gourdou I., Biener E., Gussakovsky E.E., Benomar Y., Ramanujan K.V., Taouis M., Herman B., Callebaut I. (2005). Identification of the hydrophobic strand in the AB loop of leptin as major binding site III: Implications for large-scale preparation of potents recombinant human and ovine leptin antagonists. Biochem. J..

[30] Elmquist J.K., Elias C.F., Saper C.B. (1999). From lesions to leptin: Hypothalamic control of food intake and body weight. Neuron.

[31] Elias C.F., Aschkenasi C., Lee C., Kelly J., Ahima R.S., Bjorbaek C., Flier J.S., Saper C.B., Elmquist J.K. (1999). Leptin differentially regulates NPY and POMC neurons projecting to the lateral hypothalamic area. Neuron.

[32] Fekete C., Légrádi G., Mihály E., Huang Q.-H., Tatro J.B., Rand W.M., Emerson C.H., Lechan R.M. (2000). Miα-Melanocyte-stimulating hormone is contained in nerve terminals innervating thyrotropin-releasing hormone-synthesizing neurons in the hypothalamic paraventricular nucleus and prevents fasting-induced suppression of prothyrotropin-releasing hormone gene expression. J. Neurosci..

[33] Farr S.A., Banks W.A., Morley J.E. (2006). Effects of leptin on memory processing. Peptides.

[34] Karmazyn M., Purdham D.M., Rajapurohitam V., Zeidan A. (2007). Leptin as a cardiac hypertrophic factor: A potential target for therapeutics. Trends Cardiovasc. Med..

[35] Dardeno T.A., Chou S.H., Moon H.-S., Chamberland J.P., Fiorenza C.G., Mantzoros C.S. (2010). Leptin in human physiology and therapeutics. Front. Neuroendocrinol..

[36] Janečková R. (2001). The role of leptin in human physiology and pathophysiology. Physiol. Res..

[37] Huang L., Li C. (2000). Leptin: A multifunctional hormone. Cell Res..

[38] Ducy P., Amling M., Takeda S., Priemel M., Schilling A.F., Beil F.T., Shen J., Vinson C., Rueger J.M., Kaesenty G. (2000). Leptin inhibits bone formation through a hypothalamic relay: A central control of bone mass. Cell.

[39] Ceddia R.B., Koistinen H.A., Zierath J.R., Sweeney G. (2002). Analysis of paradoxical observations on the association between leptin and insulin resistance. FASEB J..

[40] Gainsford T., Willson T.A., Metcalf D., Handman E., McFarlane C., Ng A., Nicola N.A., Alexander W.S., Hilton D.J. (1996). Leptin can induce proliferation, differentiation, and functional activation of hemopoietic cells. Proc. Natl. Acad. Sci. USA.

[41] Sierra-Honigmann M.R., Nath A.K., Muraki C., Garcia-Candeña G., Papapetropoulos A., Sessa W.C., Madge L.A., Schechner J.S., Schwabb M.B., Polverini P.J. (1998). Biological action of leptin as an angiogenic factor. Science.

[42] Murad A., Nath A.K., Cha S.-T., Demir E., Flores-Riveros J., Sierra-Honigmann M.R. (2003). Leptin is an autocrine/paracrine regulator of wound healing. FASEB J..

[43] Loffreda S., Yang S.Q., Lin H.Z., Karp C.L., Brengman M.L., Wang D.J., Klein A.S., Bulkley G.B., Bao C., Noble P.W. (1998). Leptin regulates proinflammatory immune responses. FASEB J..

[44] Münzberg H., Morrison C.D. (2015). Structure, production and signaling of leptin. Metabolism.

[45] Olea-Flores M., Juárez-Cruz J.C., Mendoza-Catalán M.A., Padilla-Benavides T., Navarro-Tito N. (2018). Signaling pathways induced by leptin during Epithelial⁻Mesenchymal Transition in breast cancer. Int. J. Mol. Sci..

[46] Andò S., Gelsomino L., Panza S., Giordano C., Bonofiglio D., Barone I., Catalano S. (2019). Obesity, Leptin and breast cancer: Epidemiological evidence and proposed mechanisms. Cancers.

[47] Ray A., Cleary M.P. (2017). The potential role of leptin in tumor invasion and metastasis. Cytokine Growth Factor Rev..

[48] Wu C., Wang L., Chen W., Zou S., Yang A. (2017). Associations between body mass index and lymph node metastases of patients with papillary thyroid cancer: A retrospective study. Medicine.

[49] O’Sullivan J., Lysaght J., Donohoe C.L., Reynolds J.V. (2018). Obesity and gastrointestinal cancer: The interrelationship of adipose and tumour microenvironments. Nat. Rev. Gastroenterol. Hepatol..

[50] Comninos A.N., Jayasena C.N., Dhillo W.S. (2014). The relationship between gut and adipose hormones, and reproduction. Hum. Reprod. Update.

[51] González R.R., Caballero-Campo P., Jasper M., Mercader A., Devoto L., Pellicer A., Simon C. (2000). Leptin and leptin receptor are expressed in the human endometrium and endometrial leptin secretion is regulated by the human blastocyst. J. Clin. Endocrinol. Metab..

[52] Sanchez-Garrido M.A., Tena-Sempere M. (2013). Metabolic control of puberty: Roles of leptin and kisspeptins. Horm. Behav..

[53] Prokop J., Duff R.J., Ball H.C., Copeland D.L., Londraville R.L. (2012). Leptin and leptin receptor: Analysis of a structure to function relationship in interaction and evolution from humans to fish. Peptides.

[54] Madej T., Boguski M.S., Bryant S.H. (1995). Threading analysis suggests that the obese gene product may be a helical cytokine. FEBS Lett..

[55] Tartaglia L., Dembski M., Weng X., Deng N., Culpepper J., Devos R., Richards G.J., Campfield L.A., Clark F.T., Deeds J. (1995). Identification and expression cloning of a leptin receptor, OB-R. Cell.

[56] Friedman J.M. (2019). Leptin and the endocrine control of energy balance. Nat. Metab..

[57] Garofalo C., Surmacz E. (2006). Leptin and cancer. J. Cell. Physiol..

[58] Liefers S.C., Veerkamp R.F., Te Pas M.F.W., Chilliard Y., van der Lende T. (2005). Genetics and physiologicy of leptin in periparturient dairy cows. Domest. Anim. Endocrinol..

[59] Lundin A., Rohdahl H., Walum E., Wilcke M. (2000). Expression and intracellular localization of leptin receptor long isoform- GFP chimera. Biochim. Biophys. Acta.

[60] Poetsch M.S., Stran A., Guan K. (2020). Role of leptin in cardiovascular diseases. Front. Endocrinol..

[61] Haniu M., Arakawa T., Bures E.J., Young Y., Hui J.O., Rohde M.F., Welcher A.A., Horan T. (1998). Human leptin receptor. Determination of disulfide structure and N-glycosylation sites of the extracellular domain. J. Biol. Chem..

[62] Oswal A., Yeo G. (2010). Leptin and the control of body weight: A review of its diverse central targets, signaling mechanism, and role in the pathogenesis of obesity. Obesity.

[63] Wauman J., Tavernier J. (2011). Leptin receptor signaling: Pathway to leptin resistance. Front. Biosci..

[64] Wauman J., Zabeau L., Tavernier J. (2017). The leptin receptor complex: Heavier than expected?. Front. Endocrinol..

[65] Frühbeck G. (2006). Intracellular signaling pathway activates by leptin. Biochem. J..

[66] Tartaglia L.A. (1997). The leptin receptor. J. Biol. Chem..

[67] Peelman F., Zabeau L., Moharanna K., Savvides S.N., Tavernier J. (2014). Insights into signaling assemblies of leptin receptor. J. Endocrinol..

[68] Kloek C., Haq A.K., Dunn S.L., Lavery H.J., Banks A.S., Myers M.G. (2002). Regulation of Jak kinases by intracellular leptin receptor sequences. J. Biol. Chem..

[69] Fei H., Okano H.J., Li C., Lee G.-H., Zhao C., Darnell R., Friedman J.M. (1997). Anatomic localization of alternatively spliced leptin receptors (Ob-R) in mouse brain and other tissues. Proc. Natl. Acad. Sci. USA.

[70] Hileman S.M., Tornøe J., Flier J.S., Bjørbaek C. (2000). Transcellular transport of leptin by the short leptin receptor isoform ObRa in Madin-Darby Canine Kidney cells. Endocrinology.

[71] Wauman J., De Ceuninck L., Vanderroost N., Lievens S., Tavernier J. (2011). RNF41 (Nrdp1) controls type 1 cytokine receptor degradation and ectodomain shedding. J. Cell Sci..

[72] Margetic S., Gazzola C., Pegg G.G., Hill R.A. (2002). Leptin: A review of its peripheral actions and interactions. Int. J. Obes. Relat. Metab. Disord..

[73] Park H.-K., Ahima R.S. (2014). Leptin signaling. F1000Prime Rep..

[74] Baumann H., Morella K.K., White D.W., Dembski M., Bailon P.S., Kim H., Lai C.F., Tartaglia L.A. (1996). The full-length leptin receptor has signaling capabilities of interleukin 6-type cytokine receptors. Proc. Natl. Acad. Sci. USA.

[75] Banks A.S., Davis S.M., Bates S.H., Myers M.G. (2000). Activation of downstream signals by the long form of the leptin receptor. J. Biol. Chem..

[76] Hekerman P., Zeidler J., Bamberg-Lemper S., Knobelspies H., Lavens D., Tavernier J., Joost H.-G., Becker W. (2005). Pleiotropy of leptin receptor signalling is defined by distinct roles of the intracellular tyrosines. FEBS J..

[77] Zhou Y., Rui L. (2013). Leptin signaling and leptin resistance. Front. Med..

[78] Gong Y., Ishida-takahashi R., Villanueva E.C., Fingar D.C., Münzberg H., Myers M.G. (2007). The long form of the leptin receptor regulates STAT5 and ribosomal protein S6 via alternate mechanisms. J. Biol. Chem..

[79] Bjørbaek C., El-Haschimi K., Frantz J.D., Flier J.S. (1999). The role of SOCS-3 in leptin signaling and leptin resistance. J. Biol. Chem..

[80] Gurzov E.N., Stanley W.J., Pappas E.G., Thomas H.E., Gough D.J. (2016). The JAK/STAT pathway in obesity and diabetes. FEBS J..

[81] Bjørbaek C., Buchholz R.M., Davis S.M., Bates S.H., Pierroz D.D., Gu H., Neel B.G., Myers M.G., Flier J.S. (2001). Divergent roles of SHP-2 in ERK activation by leptin receptors. J. Biol. Chem..

[82] Li C., Friedman J.M. (1999). Leptin receptor activation of SH2 domain containing protein tyrosine phosphatase 2 modulates Ob receptor signal transduction. Proc. Natl. Acad. Sci. USA.

[83] Torii S., Nakayama K., Yamamoto T., Nishida E. (2004). Regulatory mechanisms and function of ERK MAP kinases. J. Biochem..

[84] Bjorbak C., Lavery H.J., Bates S.H., Olson R.K., Davis S.M., Flier J.S., Myers M.G. (2000). SOCS3 mediates feedback inhibition of the leptin receptor via Tyr. J. Biol. Chem..

[85] You J., Yu Y., Jiang L., Li W., Yu X., Gonzalez L., Yang G., Ke G.Y.Z., Li W., Li C.L. (2010). Signaling through Tyr985 of leptin receptor as an age/diet-dependent switch in the regulation of energy balance. Mol. Cell. Biol..

[86] Vanhaesebroeck B., Waterfield M.D. (1999). Signaling by distinct classes of phosphoinositide 3-kinases. Exp. Cell Res..

[87] Niswender K.D., Schwartz M.W. (2003). Insulin and leptin revisited: Adiposity signals with overlapping physiological and intracellular signaling capabilities. Front Neuroendocr..

[88] Unger R.H. (2004). The hyperleptinemia of obesity-regulator of caloric surpluses. Cell.

[89] Minokoshi Y., Kim Y.-B., Peroni O.D., Fryer L.G.D., Müller C., Carling D. (2002). Leptin stimulates fatty-acid oxidation by activating AMP-activated protein kinase. Nature.

[90] Leggio A., Catalano S., De Marco R., Barone I., Andò S. (2014). Therapeutic potential of leptin receptor modulators. Eur. J. Med. Chem..

[91] Zabeau L., Peelman F., Tavernier J. (2014). Antagonizing leptin: Current status and future directions. Biol. Chem..

[92] Guo S., Lui M., Torroella-Kouri M., Gonzalez-Perez R.R. (2012). Oncogenic role and therapeutic target of leptin signaling in breast cancer and cancer stem cells. Biochim. Biophys. Acta.

[93] Coroniti R., Farjo R., Nuno D.J., Otvos L., Scolaro L., Surmacz E. (2016). Designer leptin receptor antagonist Allo-aca inhibits VEGF effects in ophthalmic neoangiogenesis models. Front. Mol. Biosci..

[94] Surmacz E., Otvos L. (2015). Molecular targeting of obesity pathways in cancer. Horm. Mol. Biol. Clin Investig..

[95] Panza S., Gelsomino L., Malivindi R., Rago V., Barone I., Giordano C., Giordano F., Leggio A., Comandè A., Liguori A. (2019). Leptin receptor as a potential target to inhibit human testicular seminoma growth. Am. J. Pathol..

[96] Andò S., Catalano S. (2011). The multifactorial role of leptin in driving the breast cancer microenvironment. Nat. Rev. Endocrinol..

[97] Crean-Tate K.K., Reizes O. (2018). Leptin regulation of cancer stem cells in breast and gynecologic cancer. Endocrinology.

[98] Gorska E., Popki K., Stelmaszczyk-Emmel A., Ciepiela O., Kucharska A., Wasik M. (2010). Leptin receptors. Eur. J. Med. Res..

[99] Frankenberry K.A., Skinner H., Somasundar P., McFadden D.W., Vona-Davis L.C. (2006). Leptin receptor expression and cell signaling in breast cancer. Int. J. Oncol..

[100] Choi J.-H., Choi K.-C., Auersperg N., Leung P.C.K. (2004). Overexpression of follicle-stimulating hormone receptor activates oncogenic pathways in preneoplastic ovarian surface epithelial cells. J. Clin. Endocrinol. Metab..

[101] Gonzalez R.R., Cherfils S., Escobar M., Yoo J.H., Carino C., Styer A.K., Sullivan B.T., Sakamoto H., Olawaiye A., Serikawa T. (2006). Leptin signaling promotes the growth of mammary tumors and increases the expression of vascular endothelial growth factor (VEGF) and its receptor type two (VEGF-R2). J. Biol. Chem..

[102] Verploegen S.A., Plaetinck G., Devos R., van der Heyden J., Guisez Y. (1997). A human leptin mutant induces weight gain in normal mice. FEBS Lett..

[103] Salomon G., Niv-Spector L., Gussakovsky E.E., Gertler A. (2006). Large-scale preparation of biologically active mouse and rat leptins and their L39A/D40A/F41A muteins which act as potent antagonists. Protein Expr. Purif..

[104] Gertler A., Solomon G. (2019). Pegylated human leptin D23L mutant—preparation and biological activity in vitro and in vivo in male ob/ob mice. Endocrinology.

[105] Brunner L., Whitebread S., Leconte I., Stricker-Krongrad A., Cumin F., Chiesi M., Levens N. (1999). A peptide leptin antagonist reduces food intake in rodents. Int. J. Obes. Relat. Metab. Disord..

[106] Niv-Spector L., Raver N., Friedman-Einat M., Grosclaude J., Gussakovsky E.E., Livnah O., Gertler A. (2005). Mapping leptin-interacting sites in recombinant leptin-binding domain (LBD) subcloned from chicken leptin receptor. Biochem. J..

[107] Shpilman M., Niv-Spector L., Katz M., Varol C., Solomon G., Ayalon-Soffer M., Boder E., Halpern Z., Elinav E., Gertler A. (2011). Development and characterization of high affinity leptins and leptin antagonists. J. Biol. Chem..

[108] Gregoraszczuk E.L., Rak A. (2015). Superactive human leptin antagonist reverses leptin-induced excessive progesterone and testosterone secretion in porcine ovarian follicles by blocking leptin receptors. J. Physiol. Pharm..

[109] Fiedor E., Gregoraszczuk E.L. (2016). The molecular mechanism of action of superactive human leptin antagonist (SHLA) and quadruple leptin mutein Lan-2 on human ovarian epithelial cell lines. Cancer Chemother Pharm..

[110] Fiedor E., Gregoraszczuk E.L. (2017). Superactive human leptin antagonist (SHLA), triple Lan1 and quadruple Lan2 leptin mutein as a promising treatment for human folliculoma. Cancer Chemother. Pharm..

[111] Fiedor E., Zajda K., Gregoraszczuk E.L. (2018). Leptin receptor antagonists’ action on HDAC expression eliminating the negative effects of leptin in ovarian cancer. Cancer Genom. Proteom..

[112] Otvos L. (2019). Potential leptin receptor response modifier peptides. Aust. J. Chem..

[113] Miyoshi Y., Funahashi T., Tanaka S., Taguchi T., Tamaki Y., Shimomura I., Noguchi S. (2006). High expression of leptin receptor mRNA in breast cancer tissue predicts poor prognosis for patients with high, but not low, serum leptin levels. Int. J. Cancer.

[114] Rene Gonzalez R., Watters A., Xu Y., Singh U.P., Mann D.R., Rueda B.R., Penichet M. (2009). Leptin-signaling inhibition results in efficient anti-tumor activity in estrogen receptor positive or negative breast cancer. Breast Cancer Res..

[115] Gonzalez R.R., Leavis P.C. (2003). A peptide derived from the human leptin molecule is a potent inhibitor of the leptin receptor function in rabbit endometrial cells. Endocrine.

[116] Harmon T., Harbuzariu A., Lanier V., Lipsey C.C., Kirlin W., Yang L., Gonzalez-Perez R.R. (2017). Nanoparticle-linked antagonist for leptin signaling inhibition in breast cancer. World J. Clin. Oncol..

[117] Harbuzariu A., Rampoldi A., Daley-Brown D.S., Candelaria P., Harmon T.L., Lipesey C.C., Beech D.J., Quarshie A., Ilies G.O., Gonzalez-Perez R.R. (2017). Leptin-Notch signaling axis is involved in pancreatic cancer progression. Oncotarget.

[118] Otvos L., Terrasi M., Cascio S., Cassone M., Abbadessa G., De Pascali F., Scolaro L., Knappe D., Stawikowski M., Cidic P. (2008). Development of a pharmacologically improved peptide agonist of the leptin receptor. Biochim. Biophys. Acta.

[119] Otvos L., Kovalszky I., Riolfi M., Olah J., Sztodola A., Nama K., Molino A., Piubello Q., Wade J.D., Surmacz E. (2011). Efficacy of a leptin receptor antagonist peptide in a mouse model of triple-negative breast cancer. Eur. J. Cancer.

[120] Otvos L., Kovalszky I., Scolaro L., Sztodola A., Olah J., Cassone M., Knappe D., Hoffmann R., Lovas S., Hatfield M.P.D. (2011). Peptide-based leptin receptor antagonists for cancer treatment and appetite regulation. Pept. Sci..

[121] Otvos L., Vetter S.W., Koladia M., Knappe D., Schmidt R., Ostorhazi E., Kovalszky I., Bionda N., Cudic P., Sumacz E. (2014). The designer leptin antagonist peptide Allo-aca compensates for short serum half-life with very tight binding to the receptor. Amino Acids.

[122] Beccari S., Kovalszky I., Wade J.D., Otvos L., Surmacz E. (2013). Designer peptide antagonist of the leptin receptor with peripheral antineoplastic activity. Peptides.

[123] Catalano S., Leggio A., Barone I., De Marco R., Gelsomino L., Campana A., Malivindi R., Panza S., Giordano C., Liguori A. (2015). A novel leptin antagonist peptide inhibits breast cancer growth in vitro and in vivo. J. Cell. Mol. Med..

[124] Giordano C., Chemi F., Panza S., Barone I., Bonofiglio D., Lanzino M., Cordella A., Campana A., Hasjim A., Rizza P. (2016). Leptin as a mediator of tumor-stromal interactions promotes breast cancer stem cell activity. Oncotarget.

[125] Giordano C., Gelsomino L., Barone I., Panza S., Augimeri G., Bonofiglio D., Rovito D., Naimo G.D., Leggio A., Catalano S. (2019). Leptin modulates exosome biogenesis in breast cancer cells: An additional mechanism in cell-to-cell communication. J. Clin. Med..

[126] Gelsomino L., Giordano C., La Camera G., Sisci D., Marsico S., Campana A., Tarallo R., Rinaldi A., Fuqua S., Leggio A. (2020). Leptin signaling contributes to aromatase inhibitor resistant breast cancer cell growth and activation of macrophages. Biomolecules.

[127] Panza S., Russo U., Giordano F., Leggio A., Barone I., Bonofiglio D., Gelsomino L., Malivindi R., Conforti F.L., Naimo G.D. (2020). Leptin and Notch Signaling Cooperate in Sustaining Glioblastoma Multiforme Progression. Biomolecules.

[128] Grasso P., Rozhavskaya-Arena M., Leinung M.C., Lee D.W. (2001). [D-LEU-4]-OB3, a synthetic leptin agonist, improves hyperglycemic control in C57BL/6J ob/ob mice. Regul. Pept..

[129] Chin Y.-T., Wang L.-M., Hsieh M.-T., Shih Y.-J., Nana A.W., Changou C.A., Yang Y.-C.S.H., Chiu H.-C., Fu E., Daviz P.J. (2017). Leptin OB_3_ peptide suppresses leptin-induced signaling and progression in ovarian cancer cells. J. Biomed. Sci..

[130] Yang Y.C., Chin Y.-T., Hsieh M.-T., Lai H.-Y., Ke C.-C., Crawford D.R., Lee O.K., Fu E., Mousa S.A., Grasso P. (2016). Novel leptin OB_3_ peptide-induced signaling and progression in thyroid cancers: Comparison with leptin. Oncotarget.

[131] Ho Y., Wang S.-H., Chen Y.-R., Li Z.-L., Chin Y.-T., Yang Y.-C.S.H., Wu Y.-H., Su K.-W., Chiu H.-C., Crewford D.R. (2019). Leptin-derived peptides block leptin-induced proliferation by reducing expression of pro-inflammatory genes in hepatocellular carcinoma cells. Food Chem. Toxicol..

[132] Anderson B.M., Jacobson L., Novakovic Z.M., Grasso P. (2017). Oral delivery of [D-Leu-4]-OB3 and MA-[D-Leu-4]-OB3, synthetic peptide leptin mimetics: Immunofluorescent localization in the mouse hypothalamus. Brain Res..

[133] Hirschstein Z., Vanga G.R., Wang G., Novakovic Z.M., Grasso P. (2020). MA-[D-Leu-4]-OB_3_, a small molecule synthetic peptide leptin mimetic, improves episodic memory, and reduces serum levels of tumor necrosis factor-alpha and neurodegeneration in mouse models of Type 1 and Type 2 Diabetes Mellitus. Biochim. Biophys Acta Gen. Subj..

[134] Fazeli M., Zarkesh-Esfahani H., Wu Z., Maamra M., Bidlingmaier M., Pockley A.G., Watson P., Matarese G., Strasburger C.J., Ross R.J.M. (2006). Identification of a monoclonal antibody against the leptin receptor that acts as an antagonist and blocks human monocyte and T cell activation. J. Immunol. Methods.

[135] Fusco R., Galgani M., Procaccini C., Franco R., Pirozzi G., Fucci L., Laccetti P., Matarese G. (2010). Cellular and molecular crosstalk between leptin receptor and estrogen receptor-α in breast cancer: Molecular basis for a novel therapeutic setting. Endocr. Relat. Cancer.

[136] Carpenter B., Hemsworth G.R., Wu Z., Maamra M., Strasburger C.J., Ross R.J., Artymiuk P.J. (2012). Structure of the human obesity receptor leptin-binding domain reveals the mechanism of leptin antagonism by a monoclonal antibody. Structure.

[137] Munikumar M., Krishna V.S., Reddy V.S., Rajeswari B., Sriram D., Rao M.V. (2018). In silico design of small peptides antagonist against leptin receptor for the treatment of obesity and its associated immune-mediated diseases. J. Mol. Graph. Model.

[138] Lei M.M., Wei C.K., Chen Z., Yosefi S., Zhu H.X., Shi Z.D. (2018). Anti-leptin receptor antibodies strengthen leptin biofunction in growing chickens. General and Comparative Endocrinology.

[139] Zabeau L., Wauman J., Dam J., Van Lint S., Burg E., De Geest J., Rogge E., Silva A., Jockers R., Tavernier J. (2019). A novel leptin receptor antagonist uncouples leptin’s metabolic and immune functions. Cell Mol. Life Sci..

[140] Muyldermans S. (2013). Nanobodies: Natural single-domain antibodies. Annu. Rev. Biochem..

[141] Zabeau L., Verhee A., Catteeuw D., Faes L., Seeuws S., Decruy T., Elewaut D., Peelman F., Tavernier J. (2011). Selection of non-competitive leptin antagonists using a random nanobody-based approach. Biochem. J..

[142] McMurphy T., Xiao R., Magee D., Slater A., Zabeau L., Tavernier J., Cao L. (2014). The anti-tumor activity of a neutralizing nanobody targeting leptin receptor in a mouse model of melanoma. PLoS ONE.

[143] Arezumand R., Alibakshi A., Ranjbari J., Ramazani A., Muyldermans S. (2017). Nanobodies as novel agents for targeting angiogenesis in solid cancers. Front. Immunol..

